# Circulating levels of cytokines and risk of cardiovascular disease: a Mendelian randomization study

**DOI:** 10.3389/fimmu.2023.1175421

**Published:** 2023-05-25

**Authors:** Tao Wei, Zhanfang Zhu, Lin Liu, Bo Liu, Min Wu, Wei Zhang, Qianwei Cui, Fuqiang Liu, Ronghuai Zhang

**Affiliations:** ^1^ Department of Cardiology, Shaanxi Provincial People’s Hospital, Xi’an, China; ^2^ Department of General Internal Medicine, Xi’an Jiaotong University Hospital, Xi’an, China; ^3^ Shaanxi Provincial Key Laboratory of Infection and Immune Diseases, Shaanxi Provincial People’s Hospital, Xi’an, China

**Keywords:** circulating cytokines, cardiovascular disease, Mendelian randomization, *cis*-quantitative trait locus, European

## Abstract

**Background:**

Epidemiological studies have linked various circulating cytokines to cardiovascular disease (CVD), which however remains uncertain whether these relationships represent causality or are due to bias. To address this question, we conducted a Mendelian randomization (MR) analysis to systematically investigate the causal effects of circulating cytokine levels on CVD development.

**Methods:**

This study leveraged the summary statistic from respective genome-wide association study (GWAS) of 47 cytokines and four types of CVD. The *cis*-quantitative trait locus (*cis*-QTL) definition, derived from a GWAS meta-analysis comprising 31,112 participants of European descent, served as instruments for cytokines. A two-sample MR design was employed, followed by comprehensive sensitivity analyses to validate the robustness of results.

**Results:**

The results of inverse-variance weighted method using *cis*-protein QTL (*cis*-pQTL) instruments, showed the causal effects of four cytokines (i.e., IL-1ra, MCSF, SeSelectin, SCF) on the risk of coronary artery disease (CAD). We also identified causal relationships between two cytokines (i.e., IL-2ra, IP-10) and heart failure (HF), as well as two cytokines (i.e., MCP-3, SeSelectin) and atrial fibrillation (AF), after controlling for false discovery rate (FDR). The use of *cis*-expression QTL (*cis*-eQTL) revealed additional causal associations between IL-1a, MIF and CAD, between IL-6, MIF, and HF, as well as between FGFBasic and AF. No significant sign was survived for stroke with FDR applied. Results were largely consistent across sensitivity analyses.

**Conclusion:**

The present study provides supportive evidence that genetic predisposition to levels of certain cytokines causally affects the development of specific type of CVD. These findings have important implications for the creation of novel therapeutic strategies targeting these cytokines as a means of preventing and treating CVD.

## Introduction

Cardiovascular disease (CVD), a cluster of disorders that impact the heart and/or blood vessels, is a foremost cause of death and disability worldwide. In 2019, it is estimated that 17.9 million deaths were attributed to CVD, ranking the first leading cause of all global death (1). CVD has a complex etiology and often develops over decades before an obvious symptomatic event. Early intervention is vital to reduce morbidity and mortality from CVD, which would produce a far-reaching influence on the public health burden. Therefore, improved understanding of the causal effect of different risk factors, especially at the microscale and molecular levels, can refine prevention strategies and enable novel targets for therapeutic intervention in CVD.

Cytokines act a crucial part in regulating the inflammatory response, altering vasoconstriction and impeding endothelium-dependent vasodilation, and therefore, they may offer potential targets for preventing CVD (2). Extensive epidemiological evidence has documented strong associations between cytokines and CVD. For instance, a meta-analysis comprising 29 cohort studies demonstrated that several cytokines, such as interleukin-6 (IL-6), IL-18, and tumour necrosis factor alpha (TNF-α), each were associated with the risk of developing coronary artery disease (CAD), in an approximately log-linear manner, independent of traditional risk factors (3). Another study involving 17,180 individuals found the positive relationship of circulating levels of monocyte chemoattractant protein-1 (MCP-1) with long-term risk of stroke (4). However, classical observational designs are prone to reverse causation and confounding that hinder causal inference, and conducting clinical trials on cytokine interventions are challenging.

Mendelian randomization (MR) is a robust technology that can address the limitations accompanying observational studies mentioned above and provides the highest level of evidence hierarchy other than randomized controlled trials by leveraging genetic variants as instrumental variables (IVs) (5). This method, when certain assumptions are satisfied, could determine causality of a given exposure-outcome association. Indeed, two successive MR analyses have helped identify the causal effect of IL-6 on the development of CAD and total CVD, indicating that IL-6 blockade may serve as a novel therapeutic target (6, 7). And the extent of this benefit may be directly proportional to the degree of reduction in levels of high-sensitivity C-reactive protein (hsCRP) (6, 7). Additionally, several MR studies have also explored the causal impact of circulating cytokines on stroke (8, 9). However, the most of existing efforts were focused on one particular type of CVD or a single pro-inflammatory cytokine, leaving other cardiovascular events such as heart failure (HF) and atrial fibrillation (AF) less explored. Herein, we applied a two-sample MR framework to systematically ascertain whether there was a causality between a broad range of cytokines and the risk of CVD which encompasses CAD, HF, AF, and stroke.

## Methods

### Study design

**Figure 1:**
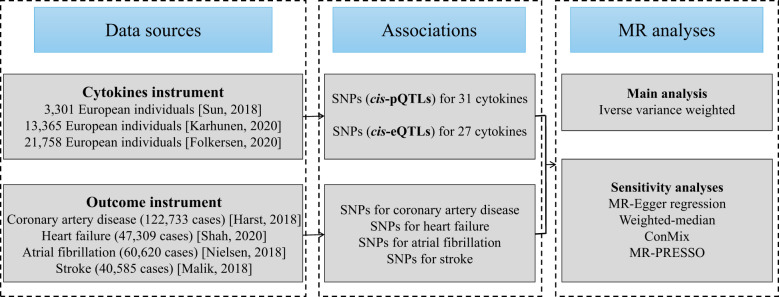
Diagram of Mendelian randomization framework in the present study.

The flowchart of the current work is outlined in **Figure 1**. To begin, we selected genetic variants as IVs for circulating cytokines. Next, latest summary statistics from the corresponding genome-wide association studies (GWASs) for CAD, HF, AF, and stroke were collected. Then, we performed a two-sample MR study with inverse-variance weighted (IVW) method as our primary analysis. Finally, a series of sensitivity analyses were followed, including MR-Egger regression, weighted-median, contamination mixture (ConMix), and MR-Pleiotropy Residual Sum and Outlier (MR-PRESSO).

### Data sources and instrumental variable selection

The present study relies on publicly available summary statistics from published studies, and therefore no additional ethical approval from the institutional review board was required. The characteristics of data used in this study are shown in **Table 1**.

**Table 1:** Characteristics of data in this study.

Phenotype	PMID	Available year	Source	Sample size	Ancestry
Exposures					
Cytokines	33067605	2020	SCALLOP Consortium	21,758	100% European
	–	2020	Karhunen et al.	13,365	100% European
	29875488	2018	INTERVAL Study	3,301	100% European
Outcomes					
Coronary artery disease	29212778	2018	CARDIoGRAMplusC4D Consortium	122,733 cases and 424,528 controls	77% European
Heart failure	31919418	2020	HERMES Consortium	47,309 cases and 930,014 controls	100% European
Atrial fibrillation	30061737	2018	Nielsen et al.	60,620 cases and 970,216 controls	100% European
Stroke	29531354	2018	MEGASTROKE Consortium	40,585 cases and 40,6111 controls	100% European

For the cytokines as exposures, we acquired the IVs from the most up-to-date GWAS meta-analysis of three independent sources (10) [i.e., the SCALLOP consortium (11), the INTERVAL study (12), and a the Northern Finland Birth Cohort 1966 (13)], which include a total of 31,112 subjects of European ancestry. Details on the methods of SNP selection and meta-analysis can be found in the original paper (10).

For the outcomes, the summary-level data for CAD (122,733 cases and 424,528 controls), HF (47,309 cases and 930,014 controls), AF (60,620 cases and 970,216 controls), and stroke (40,585 cases and 40,6111 controls) were derived from CARDIoGRAMplusC4D Consortium (14), HERMES Consortium (15), a GWAS conducted by Nielsen et al. (16), and MEGASTROKE Consortium (17), respectively. These study populations were predominantly of European descent. There is no obvious sample overlap between the GWAS meta-analyses of cytokines and four investigated cardiovascular diseases.

MR relies on three IV assumptions to ensure the accuracy of causal inferences (1): IVs must truly be associated with exposures (2), independent of confounders, and (3) affect outcomes solely through exposures, not through any other pathways. To better ensure that the selected IVs satisfy the above three assumptions, especially for the first and the third, we here used *cis* quantitative trait locus (QTLs) as IVs to enhance instrument strength, including *cis*-protein QTLs (*cis*-pQTLs, gene range ± 500kb) and *cis*-expression QTLs (*cis*-eQTLs, gene range ± 500kb). These *cis*-QTLs, located at or close to the gene of origin, naturally have a stronger correlation with the gene expression and thereafter protein concentrations than other variants. For our main analysis, we used *cis*-pQTLs as the IVs, and for complementary analysis, we used *cis*-eQTLs since *cis*-eQTLs may capture the effects of pQTLs through gene expression, although not all pQTLs are represented by eQTLs (18). Specifically, *cis*-pQTLs and *cis*-eQTLs were associated with the circulating cytokine levels and their gene expression aggregated across tissues, respectively, both met a significant threshold of 1E-4. Of note, to better balance the number and strength of instrumental variables and obtain potentially informative results, a relatively loose threshold of 1E-4 was selected as an alternative to the 5E-8 threshold used in traditional GWAS. We excluded palindromic variants with a minor allele frequency (MAF) greater than 0.05 and performed clumping by setting a pairwise linkage disequilibrium (LD) cutoff of r^2^ < 0.1. The alleles of the QTLs were harmonized between the exposure and the outcome to ensure proper alignment of effects. Detailed information on the characteristics of the QTLs used as IVs can be found in the **Supplementary Tables 1, 2**.

### Statistical analyses

We incorporated separate analyses using two different sets of IVs (*cis*-pQTL and *cis*-eQTL) to assess the links between genetically predicted circulating cytokine levels and the risk of each CVD outcome. For cases where there exists only one single QTL, the classic Wald ratio was adopted to gain MR estimates. Otherwise, the random-effects IVW model using a meta-analysis approach was performed to combine the Wald ratios of multiple QTLs to obtain overall MR estimates (19). IVW is generally considered to provide unbiased estimates of the causal effect of the exposure on the outcome, provided that the three assumptions mentioned above are met. Furthermore, we conducted sensitivity analyses using four pleiotropy- robust methods, namely MR-Egger regression (20), weighted-median method (21), ConMix (22), and MR-PRESSO (23). Different methods could yield valid results under different model assumptions with the criterion relaxed to some extent. The weighted-median estimator is capable of providing a valid estimation even when 50% IVs are invalid. The MR-Egger regression is sensitive to the presence of horizontal pleiotropy across IVs, but it depends on the Instrument Strength Independent of Direct Effect (InSIDE) assumption, where the genetic variant used as an instrument affects the outcome only through the exposure of interest and not through any other pathway that could confound the association between the exposure and the outcome. The method ConMix could estimate the causal effect with implicitly distinguishing valid and invalid IVs using an underlying mixture model. MR-PRESSO can obtain consistent causal estimates by horizontal pleiotropic outlier (if it is noted) removal. These methods rely on different assumptions to each other which are difficult to prove, and if the results from all of these different analyses are largely consistent, then the investigator can be more confident in drawing conclusions regarding causality. Moreover, the Cochran’s Q test in the IVW and the intercept from MR-Egger method were deployed to test the heterogeneity and horizontal pleiotropy, with a *P*-value < 0.05, respectively. The original paper provided information on the proportion of variance (R^2^) that each cytokine has explained by the QTLs and *F*-statistics that quantified the strength of the IVs (10). We further calculated the cumulative *F*-statistics and an *F*-statistic > 10 was considered to avoid weak instrument bias (24). The statistical power of our entire MR analyses was estimated utilizing the non-centrality parameter-based approach proposed by Brion et al. on the online tool (https://shiny.cnsgenomics.com/mRnd/) (25). Lastly, correlation analysis was done to illustrate the mutual corroboration or complementary of results under two instruments strategies.

All statistical analyses were implemented using the “TwoSampleMR” (version 0.5.6), “MendelianRandomization” (version 0.6.0), and “MR-PRESSO” (version 1.0) packages in R (version 4.1.2). To address the issue of multiple comparisons for the numerous cytokines, we performed the stratified false discovery rate (FDR) approach using Benjamini-Hochberg procedure for IVW analysis, i.e., estimating adjusted *P*-value separately for each CVD outcome (26). Additionally, we also utilized the aggregated FDR correction to complement our results. The statistical significance was defined using a threshold of adjusted *P*-value < 10%.

## Results

**Figure 2:**
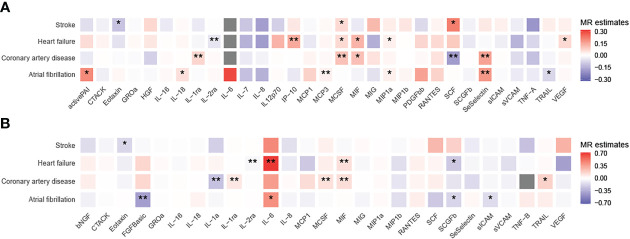
Heatmaps of the causal associations between cytokines and cardiovascular diseases. Shown are the results of the IVW method based on the **(A)** cis-pQTL and **(B)** cis-eQTL instruments. Red boxes indicate positive associations, blue boxes negative associations, and grey boxes associations for which no instrument was available. One asterisk denotes that the associations is nominally significant (*P* < 0.05). Two asterisks denote that the association withstands multiple comparison correction (stratified FDR < 10%).

**Figure 3:**
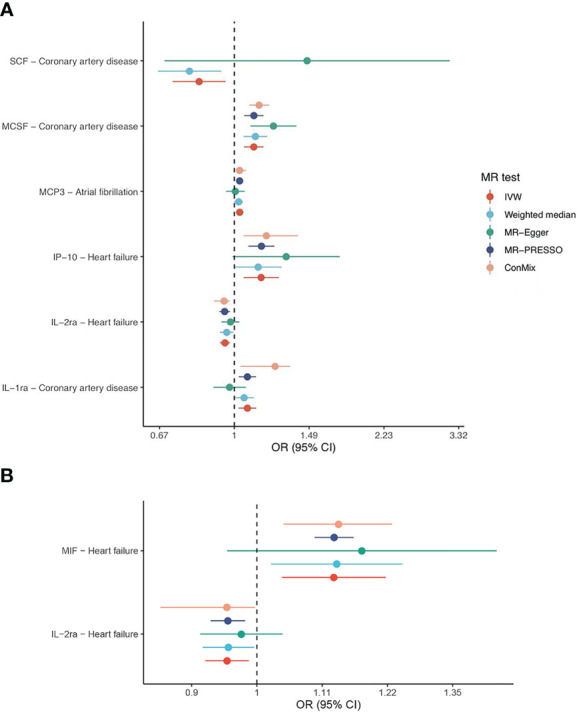
Forest plots of IVW and sensitivity analyses. Shown are the associations that withstand multiple comparison correction (stratified FDR < 10%) based on the **(A)** cis-pQTL and **(B)** cis-eQTL instruments.

To unravel the causal effect of all analyzable cytokines (shown in **Table 2**) on the risk of four types of CVD (i.e., CAD, HF, AF, stroke), two-sample MR tests were carried out. Of 35 cytokines analyzed, 31 cytokines possessed *cis*-pQTL instruments, explaining 0.1% to 28.9% of the phenotypic variance, and 27 cytokines possessed *cis*-eQTL instruments, explaining 0.04% to 13.0% of the phenotypic variance. Cumulative *F*-statistics of *cis*-pQTL for 29 out of 31 cytokines were greater than 10, proving the good strength of genetic instruments (**Table 2**). Detailed MR results for the causal relationship of interests are shown in **Supplementary Table 3** (based on the cis-pQTL instruments) and **Supplementary Table 4** (based on the cis-eQTL instruments). The visualization of all IVW results is presented in **Figure 2** and only significant IVW results (stratified FDR < 10%) with sensitivity analyses are shown in **Figure 3**. Furthermore, the results of the two correction strategies (stratified FDR vs. aggregated FDR) did not differ significantly.

**Table 2:** Instruments for each studied cytokine in the *cis*-pQTL and *cis*-eQTL analyses.

		*cis*-pQTL			*cis*-eQTL		
Cytokine	Gene	No. SNPs	R^2^	*F*-statistic	No. SNPs	R^2^	*F*-statistic
activePAI	SERPINE1	1	0.003	16	–	–	
bNGF	NGF	–	–		1	0.002	7
CTACK	CCL27	3	0.060	75	2	0.041	77
Eotaxin	CCL11	6	0.015	29	4	0.010	30
FGFBasic	FGF2	–	–		2	0.002	8
GROa	CXCL1	11	0.272	186	1	0.127	993
HGF	HGF	6	0.010	19	–	–	
IL-16	IL16	18	0.037	44	6	0.031	114
IL-18	IL18	5	0.051	71	2	0.024	86
IL-1a	IL1A	–	–		3	0.003	5
IL-1ra	IL1RN	18	0.075	79	2	0.017	165
IL-2ra	IL2RA	14	0.260	72	4	0.130	128
IL-6	IL6	1	0.002	16	1	0.001	8
IL-7	IL7	1	0.005	16	–	–	
IL-8	CXCL8	1	0.004	72	2	0.005	41
IL-12p70	IL12A	1	0.002		–	–	
IL-12p70	IL12B	1	0.002		–	–	
IP-10	CXCL10	5	0.020	35	–	–	
MCP-1	CCL2	28	0.006	4	3	0.001	8
MCP-3	CCL7	13	0.289	77	–	–	
MCSF	CSF1	13	0.049	62	3	0.018	93
MIF	MIF	2	0.019	34	5	0.020	14
MIG	CXCL9	1	0.011	41	2	0.008	15
MIP1a	CCL3	34	0.217	111	1	0.059	1194
MIP1b	CCL4	26	0.147	67	3	0.003	12
PDGFbb	PDGFB	1	0.001	18	–	–	
RANTES	CCL5	1	0.009	31	1	0.009	31
SCF	KITLG	3	0.006	30	2	0.001	7
SCGFb	CLEC11A	2	0.016	57	1	0.004	28
SeSelectin	SELE	2	0.008	91	2	0.002	7
sICAM	ICAM1	25	0.168	35	2	0.004	11
sVCAM	VCAM1	1	0.003	16	1	0.003	16
TNF-A	TNF	2	0.004	19	–	–	
TNF-B	LTA	–	–		1	0.001	5
TRAIL	TNFSF10	46	0.027	7	5	0.006	14
VEGF	VEGFA	21	0.073	105	1	0.0004	10

activePAI, active plasminogen activator inhibitor-1; bNGF, beta nerve growth factor; CTACK, cutaneous T-cell attracting chemokine; FGFBasic, basic fibroblast growth factor; GROa, growth-regulated oncogene-alpha; HGF, hepatocyte growth factor; IL, interleukin; ra, receptor antagonist; IP-10, interferon gamma-induced protein 10; MCP-1, monocyte chemotactic protein-1; MCP-3, monocyte chemotactic protein-3; MCSF, macrophage colony-stimulating factor; MIF, macrophage migration inhibitory factor; MIG, monokine induced by interferon-gamma; MIP, macrophage inflammatory protein; PDGFbb, platelet-derived growth factor BB; SCF, stem cell factor; SCGFb, stem cell growth factor beta,; SeSelectin, soluble E-selectin; sICAM, soluble intercellular adhesion molecule; sVCAM, soluble vascular cell adhesion molecule; TNF, tumour necrosis factor; TRAIL, TNF-related apoptosis inducing ligand; VEGF, vascular endothelial growth factor.

Using the *cis*-pQTL instruments in IVW analysis, genetic predicated higher levels of four cytokines had a suggestive association with an increased risk of CAD, including IL-1ra (interleukin-1 receptor antagonist, odds ratio [OR]: 1.07, 95% confidence interval [CI]: 1.02-1.12, *P*: 0.004), MCSF (macrophage colony-stimulating factor, OR: 1.11, 95% CI: 1.05-1.17, *P*: 8.88E-05), MIF (macrophage inflammatory protein, OR: 1.11, 95% CI: 1.02-1.22, *P*: 0.021), SeSelectin (soluble E-selectin, OR: 1.17, 95% CI: 1.04-1.31, *P*: 0.007), while one cytokine, SCF (stem cell factor, OR: 0.83, 95% CI: 0.72-0.95, *P*: 0.009), to a lower risk of CAD. After stratified FDR correction, the significance of MIF results was not remained. As expected, when using the *cis*-eQTL instruments and similarly estimated by IVW, most of findings from our main analysis including IL-1ra, MCSF were replicated, yet the MIF (OR: 1.13, 95% CI: 1.06-1.22, *P*: 4.15E-04) maintained notable significant relationship with CAD risk. In addition, we observed the novel evidence regarding IL-1a (OR: 0.77, 95% CI: 0.64-0.92, *P*: 0.004).

Likewise, tested by IVW method using the *cis*-pQTL instruments, six cytokines (IL-2ra, IP-10 [interferon gamma-induced protein 10], MCSF, MIF, MIP1a, and VEGF [vascular endothelial growth factor]) showed suggestive association with the risk of HF, six cytokines (activePAI [active plasminogen activator inhibitor-1], IL-18, MCP-3, MIP1a, SeSelectin, and TRAIL [TNF-related apoptosis inducing ligand]) with AF, and three cytokines (Eotaxin, MCSF, and SCF) with stroke. When considering the stratified FDR of 10% or less, only two cytokines, IL-2ra (OR: 0.95, 95% CI: 0.93-0.98, *P*: 2.93E-04) and IP-10 (OR: 1.15, 95% CI: 1.05-1.27, *P*: 0.003), reached a statistical significance (stratified FDR < 10%) for HF, as well as two cytokines, MCP-3 (OR: 1.03, 95% CI: 1.01-1.05, *P*: 0.003) and SeSelectin (OR: 1.19, 95% CI: 1.07-1.32, *P*: 0.002), for AF. No significant sign was survived for stroke after stratified FDR correction. With *cis*-eQTL instruments and stratified FDR adjustment applied, the additional associations between IL-6 (OR: 2.01, 95% CI: 1.27-3.17, *P*: 0.003), MIF (OR: 1.13, 95% CI: 1.04-1.22, *P*: 0.004) and HF, as well as FGFBasic (basic fibroblast growth factor) and AF (OR: 0.66, 95% CI: 0.5-0.84, *P*: 0.001) were captured.

Focused on the associations that withstand multiple comparison correction (stratified FDR < 10%), their corresponding sensitivity analyses indicated roughly the same estimates although several methods yield wide CIs due to less statistical power (refer to **Figure 3**). Further examination revealed little evidence of heterogeneity (majority of P-value of Cochran Q statistic > 0.05) or horizontal pleiotropy (majority of P-value of MR-Egger intercept > 0.05), as shown in the **Supplementary Tables 5, 6**.

**Figure 4:**
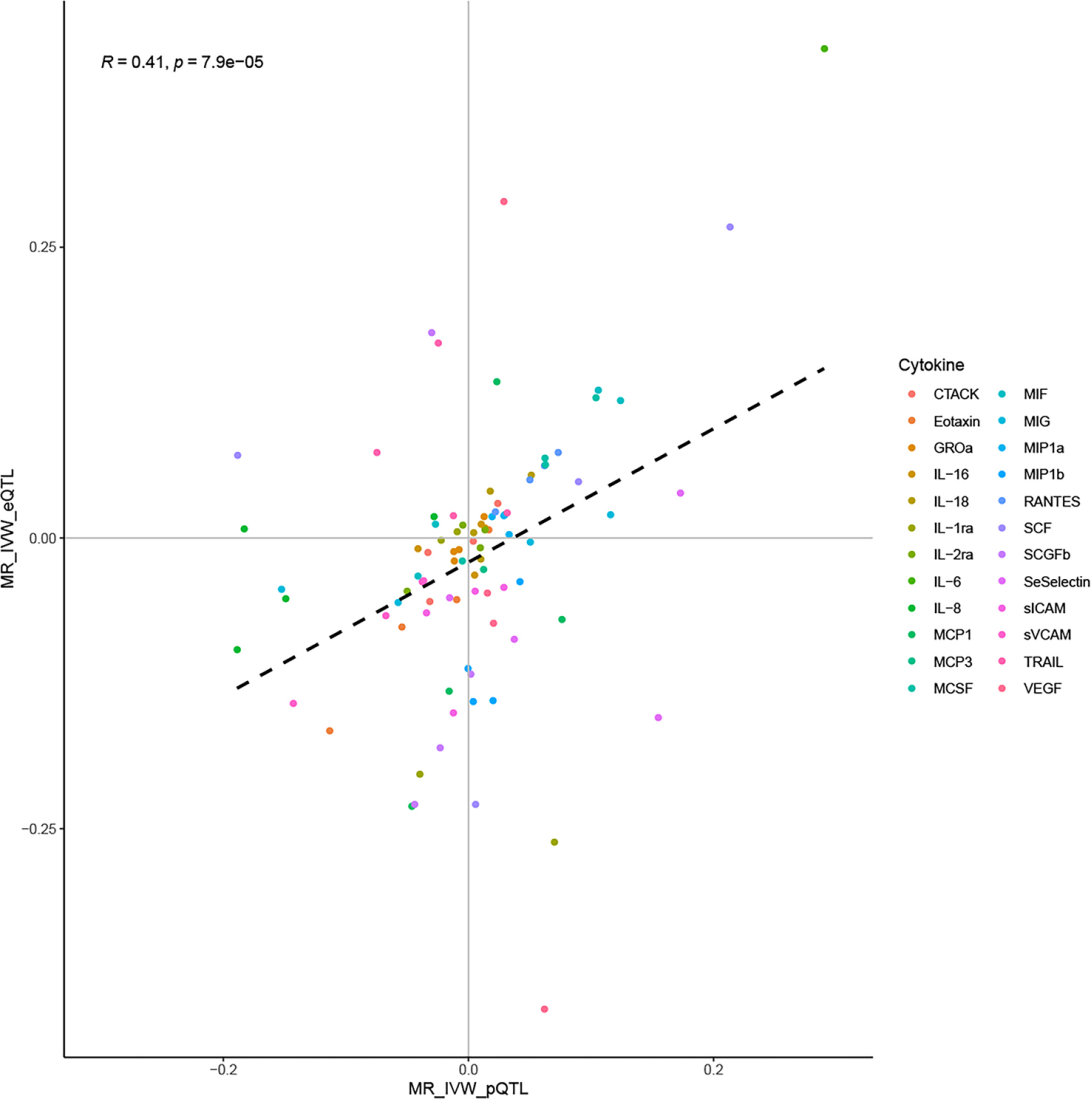
Correlation of IVW estimates using the *cis*-pQTL and *cis*-eQTL instruments.

Correlation analysis suggested a moderate correlation of IVW estimates obtained using the *cis*-pQTL and *cis*-eQTL instruments (correlation coefficient: 0.41, *P*: 7.9E-05, **Figure 4**). Their moderate correlation, combined with the biological background foreshadowed earlier, validated the rationality of using *cis*-pQTL instruments for the main analysis and *cis*-eQTL as a complement. Furthermore, sufficient statistical power was achieved in our MR study to detect the causal associations of cytokines with CVD outcomes, provided they were true. By complementing the statistical power of the two instruments, we attained a power of 100% to detect an OR of 1.2/0.83 for the majority of the associations (**Supplementary Tables 7, 8**).

## Discussion

In our MR analysis, we systematically assessed the causal roles of circulating cytokine levels in the four CVD outcomes. The results provided the strong evidence in favor of the causal relationships of genetic predicted levels of IL-1ra, MCSF, SCF, and SeSelectin with CAD, genetic predicted levels of IL-2ra and IP-10 with HF, as well as genetic predicted levels of MCP-3 and SeSelectin with AF. There was also suggestive evidence supporting causal effect of IL-1a, MIF concentrations on CAD, of IL-6, MIF concentrations on HF, as well as of FGFBasic concentrations on AF. The relationship of circulating cytokine concentrations with stroke were attenuated after correction for multiple comparisons. Using the power of molecular genetic markers as IVs, particularly through the use of both pQTL and eQTL instruments, our study was able to overcome potential biases and confounders that arise in observational studies. Our results support the hypothesis that manipulating cytokine levels may represents a promising therapeutic strategy for CVD.

Our findings strengthened or extended existing observational evidence, pointing to an essential role played by certain cytokines, including IP-10, IL-6, IL-1ra, and SCF, in the development of CVD. A study combining data from two prospective cohorts suggested that higher concentration of IP-10 was related to the elevated risk of HF but not CAD or stroke, which largely aligns with our own results (27). In addition, our findings regarding IL-6, based on the eQTL instruments, in relation to HF was in line with the results from a case-cohort study (28). A meta-analysis of six population-based cohorts reported that serum IL-1ra level was positively related to the risk of total CVD (29). A 19.2-year follow-up study with 4,742 participants demonstrated that individuals with high levels of SCF have a decreased risk of cardiovascular events (30). Our results add further specificity to these findings, indicating that the IL-1ra and SCF were more likely to represent the causal factors for CAD risk. This conclusion was also confirmed by a recent MR study that used a different set of IVs (31). Nevertheless, the aforementioned MR analysis gave the evidence for the involvement of IL-6 on CAD and AF, which was also consistent with the findings from two previous MR studies that we foreshadowed in the introduction section (6, 7). Due to the limited availability of valid pQTLs for IL-6 and a single SNP as the only eQTL instrument, our study may be underpowered to fully establish its relationship with CVD outcomes. Georgakis et al. found that genetic predicted circulating levels of MCP-1 was positively associated with stroke risk (8), while our study failed to capture this signal. This may be due to different sources of IVs (three independent cohorts v.s. two independent cross-sectional surveys) (32) and different selection criteria (*P* < 1e-4 v.s. FDR < 5%). More studies are needed to further explore. Notably, our study offered some novel insights regarding MCP-3, MCSF, and SeSelectin, which have not been previously found or minimally explored in direct relation to CVD.

Cytokines may have both direct and indirect effects on the cardiovascular system. Direct effects include alterations in the function of the heart and blood vessels such as increased heart rate, reduced blood flow, and changed blood pressure regulation (33, 34). The relationship between cytokines and CVD is complex and multilayered, with the key indirect mechanism mainly being inflammation and oxidative stress (2, 35). Inflammatory cytokines like TNF and IL-6 activate immune cells, such as monocytes and macrophages. On the one hand, activated macrophages release various inflammatory molecules and reactive oxygen species (ROS) which lead to inflammation, oxidative stress, and ultimately, endothelial dysfunction (36). On the other hand, this accumulation of immune cells and other degenerative material in the inner layer of artery walls could lead to the development of atheroma, contributing to cardiovascular events (37). More seriously, oxidative stress and inflammation have a mutually reinforcing positive feedback loop (38, 39). In contrast, anti-inflammatory cytokines like IL-1ra, which was also supported by our study, has ability to block inflammatory signals from IL-1 by binding to the IL-1 receptor (40). In addition, thrombosis caused by platelet activation and increased heart rate and blood pressure caused by adrenergic activation were also believed to be potential causative pathways induced by cytokines in the development of CVD.

One main strength of the current study is the broad scope of cytokines that we covered, as well as the substantial sample size for each trait of interest that we used, especially for cytokines, which is larger than previously used GWAS (31,112 v.s. 8,293) (32). Another important strength is the utilization of QTLs as IVs, which are in close proximity to the encoding gene region, minimizing the likelihood of horizontal pleiotropy (41). Several limitations should be acknowledged. Firstly, as mentioned early, although a relatively lenient threshold was applied, the limited number of instruments for several cytokines, such as IL-6 and/or MIF, due to the *cis*-instrument definition approach, may result in a less statistical power, especially for MR sensitivity analysis which requires a higher number of instruments. Additionally, because of the high correlation between cytokines, particularly within a category, such as the ILs family, as well as correlations among four types of CVD, the naive multiple comparison adjustment may be excessive, further affecting the false negative. We hereby reminded that even in cases of negative results, complete exclusion of causality cannot be ensured and thus such results should be interpreted with caution. Secondly, the expression of certain cytokines can be influenced by age and the changing external setting, such as a bimodal curve that has been described for IL-1ra expression throughout the life stage (42). The estimates of a lifetime effect of cytokines on CVD provided by MR may not deliver much clinical meaningful for age-specific interventions. There may be non-liner effects or interactions between cytokines that are not captured by the present study. An age-specific MR analysis especially with individual-level data was warranted in future endeavors. Thirdly, even though a wide panel of cytokines was investigated, some other important kinds of cytokines like IL-1β (43) and IL-10 (44), known for their direct role in targeting inflammation in atherosclerosis, were not analyzed in our study due to the lack of available QTL instruments for these cytokines. Lastly, due to the inaccessibility of the full summary statistics for cytokines, we were unable to conduct the colocalization analysis, which are valuable in strengthening the observed MR associations in helping identify associations that may have arisen due to confounding by LD. Further studies are warranted to strengthen our findings with colocalization when full summary statistics for cytokines are available.

## Conclusion

To conclude, based on innovative IVs that incorporate gene expression relevance and large genetic association data, this MR study comprehensively examined the causal influence of circulating cytokine levels on four major CVDs. Our MR study provides robust evidence that the levels of certain cytokines were associated with the development of CVD and highlights the importance of considering cytokines as potential targets for the prevention and management of CVD. Further research, if possible, clinical trials, are necessary to validate these findings and delve into the underlying biological mechanisms specifically at the specific cytokine level.

## Data availability statement

The original contributions presented in the study are included in the article/**Supplementary material**. Further inquiries can be directed to the corresponding author.

## Ethics statement

Ethical review and approval was not required for the study on human participants in accordance with the local legislation and institutional requirements. The patients/participants provided their written informed consent to participate in this study.

## Author contributions

RZ and TW were the major contributors in conceptualisation. TW, ZZ and LL analyzed the data. ZZ, BL, MW, WZ, QC, and FL verified the correctness of the data. TW, ZZ and RZ were major contributors in writing the manuscript. All authors contributed to the article and approved the submitted version.
